# TNF-α Orchestrates Experience-Dependent Plasticity of Excitatory and Inhibitory Synapses in the Anterior Piriform Cortex

**DOI:** 10.3389/fnins.2022.824454

**Published:** 2022-04-26

**Authors:** Anni Guo, Chunyue Geoffrey Lau

**Affiliations:** ^1^Department of Neuroscience, City University of Hong Kong, Kowloon, Hong Kong SAR, China; ^2^Shenzhen Research Institute, City University of Hong Kong, Shenzhen, China

**Keywords:** GABAergic interneurons, excitation and inhibition balance, cytokines, glial cells, homeostatic plasticity

## Abstract

Homeostatic synaptic plasticity, which induces compensatory modulation of synapses, plays a critical role in maintaining neuronal circuit function in response to changing activity patterns. Activity in the anterior piriform cortex (APC) is largely driven by ipsilateral neural activity from the olfactory bulb and is a suitable system for examining the effects of sensory experience on cortical circuits. Pro-inflammatory cytokine tumor necrosis factor-α (TNF-α) can modulate excitatory and inhibitory synapses, but its role in APC is unexplored. Here we examined the role of TNF-α in adjusting synapses in the mouse APC after experience deprivation via unilateral naris occlusion. Immunofluorescent staining revealed that activity deprivation increased excitatory, and decreased inhibitory, synaptic density in wild-type mice, consistent with homeostatic regulation. Quantitative RT-PCR showed that naris occlusion increased the expression of Tnf mRNA in APC. Critically, occlusion-induced plasticity of excitatory and inhibitory synapses was completely blocked in the Tnf knockout mouse. Together, these results show that TNF-α is an important orchestrator of experience-dependent plasticity in the APC.

## Introduction

For all sensory systems, a key function is to have faithful representation of the stimulus while maintaining the flexibility of adding associative information to it. The anterior piriform cortex (APC) is a primary sensory cortex one synapse downstream of the olfactory bulb (OB), and yet has extensive recurrent connections that resemble properties in an association cortex (Poo and Isaacson, 2007; Franks et al., 2011; Wilson and Sullivan, 2011). Information represented by principal neurons in the APC is a product of the complex interactions between excitatory and inhibitory circuits. However, how these circuits are connected to the principal neurons and how they are amenable to plastic changes are unclear.

Neurons in APC can perform context-dependent retrieval and storage of memory (Haberly, 2001; Best and Wilson, 2003; Franks et al., 2011). The extensive recurrent excitatory inputs that exhibit robust long-term synaptic plasticity may contribute to the ability of APC to perform learning and memory functions (Poo and Isaacson, 2007; Franks et al., 2011). In the APC, olfactory discrimination learning modulates synaptic strength differently in the feedforward vs. feedback pathways (Cohen et al., 2015). APC sends extensive projections to OB, posterior piriform and entorhinal cortex (Behan and Haberly, 1999; Otazu et al., 2015; Mazo et al., 2017), suggesting that plasticity in APC neuron can impact both upstream and downstream brain regions.

Homeostatic plasticity can occur via overall scaling of synapses to stabilize a neural network over Hebbian type of associative plasticity (Keck et al., 2017). Accumulating evidence suggests that the interplay between glia and neurons is a crucial communication channel for homeostatic plasticity. Pro-inflammatory cytokines, such as TNF-α and interleukin 1 beta (IL-1β), are candidate molecules that regulate excitatory and inhibitory transmission in neurons (Lai et al., 2006; Stellwagen and Malenka, 2006). TNF-α has been implicated in synaptic plasticity in different brain regions through diverse mechanisms. TNF-α is required for a homeostatic component of deprivation-induced visual cortical plasticity (Kaneko et al., 2008). Mechanistically, TNF-α increases excitatory transmission while decreases inhibitory transmission in brain slices, consistent with increases of AMPAR expression and decreases of GABA_A_R expression, respectively, on the cell surface after exogenous TNF-α treatment in cultured neurons (Beattie et al., 2002; Stellwagen et al., 2005; Stellwagen and Malenka, 2006; Pribiag and Stellwagen, 2013). Therefore, TNF-α modulates the relative contribution of excitatory and inhibitory synaptic transmission and plays a critical role in balancing excitation-inhibition (E-I) of neural circuits. Olfactory experience can regulate excitatory and inhibitory transmission in the APC (Best and Wilson, 2003; Quinlan et al., 2004; Kim et al., 2006; Poo and Isaacson, 2007; Jiang et al., 2021). However, the role of TNF-α in regulating E-I ratio in APC is unexplored.

Plasticity of inhibitory neurons is a crucial mechanism for balancing E-I, which is critical for the stable representation of patterns in a network. Although it is known that inhibition in the APC is broadly tuned (Poo and Isaacson, 2009) and that principal neurons in APC receive both recurrent and feedforward inhibition (Sheridan et al., 2014; Large et al., 2016a,b), it is unclear how dynamic activation of inhibitory inputs differentially modulate APC principal neurons. Interneurons expressing the markers parvalbumin (PV) and somatostatin (SST) are two non-overlapping subtypes of interneurons with sizeable population in APC (Suzuki and Bekkers, 2010). PV and SST neurons play different roles in regulating principal neuron activity as they impart perisomatic and dendritic inhibition, respectively. We recently showed that olfactory experience selectively regulates PV but not SST inhibition (Jiang et al., 2021). However, it is unclear whether their plasticity is regulated by soluble signaling molecules. Here, we investigated the role of TNF-α signaling in regulating experience-dependent plasticity of structural E-I in APC. As neural activity in APC is mostly driven by spiking in the ipsilateral OB (Tantirigama et al., 2017), unilateral activity deprivation can illuminate the role of experience in regulating inhibitory circuits in APC. Using a combination of immunofluorescent staining and quantitative RT-PCR, we explored the effects of sensory experience deprivation (via unilateral naris occlusion or NO) on the excitatory and inhibitory synapses and neurons in WT and Tnf knockout mice. Our results reveal that experience deprivation induced plasticity of excitatory and inhibitory synapses in a manner that is consistent with homeostatic compensation. Moreover, this homeostatic regulation was completely prevented in the Tnf knockout mouse, suggesting a critical role for TNF-α in this plasticity.

## Materials and Methods

### Animals

Both male and female mice of Tnf**^–/–^** (Jackson labs, catalog # 005540) and strain-matched wild-type mice (Jackson labs, C57BL6/J) were used in this study. Tnf**^–/–^** mice were bred homozygously as described previously (Kaneko et al., 2008). All work was done in accordance with guidelines from the Animal Research Ethics Sub-Committee of City University of Hong Kong and Department of Health of Hong Kong SAR government.

### Deprivation of Olfactory Experience

Naris occlusion (NO) was performed by using an electrocauterizer (WPI) on young adult mice (postnatal day 21–28) with anesthesia 7 days before experiment. The closed nostril was checked for its complete closure by visual inspection. After confirmation of occlusion of 7 days, mice were used for microscopy or RNA isolation.

### Real-Time Quantitative PCR of mRNA Expression

To prepare RNA samples from the anterior piriform cortex (APC), total RNA was extracted and isolated by TaKaRa MiniBEST Universal RNA Extraction Kit (Cat. #9767) according to manufacturer’s instructions. For detection and quantification of mRNA, 1 μg of total RNA was used for cDNA synthesis with TaKaRa PrimeScript RT Master Mix (Perfect Real Time, Cat. #RR036A), and quantitative RT-PCR was performed with Fast SYBR-Green Master Mix (Applied Biosystems) detected by StepOne Plus RealTime PCR system (Applied Biosystems, Life Technologies Limited, Carlsbad, CA, United States). The sequences of qPCR primers used for mRNA quantification in this study were obtained from the Primer Bank, and were as follows: mouse Gad1 (gene encoding GAD67 protein) forward, CTTCTTCAGGCTCTCCCGTG; mouse Gad1 reverse, GTATTAGGATCCGCTCCCGC; mouse vglut1 forward, CAGCCCGCCTACTTTGAAGA; mouse vglut1 reverse, GTGACGACTGCGCAAAAAGT; mouse pvalb forward, ATCAAGAAGGCGATAGGAGCC; mouse pvalb reverse, GGCC AGAAGCGTCTTTGTT; mouse Tnf forward, TAGCTCCCAG AAAAGCAAGCA; mouse Tnf reverse, CCATCTTTTGGGGGA GTGCC; mouse Tnfr1 forward, GCTGTTGCCCCTGGT TATCT; mouse Tnfr1 reverse, ATGGAGTAGACTTCGGGCCT; mouse gfap forward, CAGATCCGAGAAACCAGCCT; mouse gfap reverse, ACACCTCACATCACCACGTC; mouse gapdh forward, AGGTCGGTGTGAACGGATTTG; mouse gapdh reverse, TGTAGACCATGTAGTTGAGGTCA. After 40 cycles, the Ct values were determined. To normalize the samples, ΔCt between target genes and gapdh was calculated. The fold difference in expression between open and occluded side was then determined by subtraction of the ΔCt values and termed ΔΔCt. Finally, the total change was calculated as 2^–ΔΔ*Ct*^ and the relative amount in occluded side compared with open side was deducted.

### Immunofluorescent Staining

Mice were deeply anesthetized with ketamine/xylazine mix (100 mg/kg ketamine and 19 mg/kg xylazine) prior to fixation with 4% paraformaldehyde (PFA) in a 0.1 M phosphate buffer solution (PBS; pH 7.4; 4°C) following an initial flush with ice-cold 0.1 M PBS. Brains were post-fixed overnight in PFA and cut into 50 μm thick coronal sections in 0.1 M PBS using vibratome (Leica VT1000S). Sections were incubated for 1 h with blocking solution (5% normal goat serum, 1% Triton X-100 in PBS) before incubation overnight at 4°C with primary antibodies in blocking solution [mouse anti-GAD67 antibody (EMD Millipore MAB5406, 1:1000); rabbit anti-VGLUT1 antibody (Synaptic Systems, 135302, 1:1000); rabbit anti-parvalbumin antibody (Swant, PV27, 1:1000); rabbit anti-GFAP antibody (Dako, GA524, 1:1000); rabbit anti-Iba1 antibody (Wako, 019-19741, 1:1000); mouse anti-SST antibody (Santa Cruz Biotechnology, sc-55565, 1:1000); rabbit anti-VGAT antibody (Synaptic Systems, 131002, 1:1000); rabbit anti-Homer1 antibody (Synaptic Systems, 160003, 1:1000)]. Sections were washed 3 × 10 min with PBS at RT and incubated for 1 h at room temperature in secondary antibodies [anti-rabbit or anti-mouse IgG-conjugated Alexa Fluorochrome (Jackson Immunoresearch; 1:1000), DAPI, (Santa Cruz Biotechnology, sc-3598, 1:10000)]]. Sections were washed 3 × 10 min with PBS at RT. The stained sections were mounted on slides in VectorShield mounting medium (Vector Labs) and imaged using Zeiss LSM 880 Confocal Microscope with Zeiss imaging software. Images of anterior piriform cortex were acquired with a 10×, 20× or 40× objective.

### Image Analysis

Synapse density and DAPI^+^ cell density in sublayers of anterior piriform cortex was determined from confocal-acquired images (40×) using the FIJI-ImageJ particle analyzing tool (Analyze\Analyze Particles) with synapses or cells on edge excluded after filtering background noise (Image\Adjust\Threshold). Threshold was chosen to exclude background and include structure that appears punctate after measuring gray value of background and target puncta. Maximal threshold was left at a maximum (255 for 8-bit images). Threshold was determined for each protein individually. PV^+^ neuron density in APC were quantified from confocal-acquired images (10×) using the FIJI-ImageJ particle analyzing tool, with neurons on edge excluded. Astrocyte (GFAP^+^) and microglia (Iba1^+^) densities in APC were quantified from confocal-acquired images (20×) by manual counting. Only cells co-localized with DAPI^+^ nuclei would be considered as astrocytes or microglia. GFAP or Iba1 expression covered area percentage and mean intensity level were quantified using FIJI-ImageJ analyzing tool (Analyze\Measure) after filtering background noise (Image\Adjust\ Threshold).

### Experimental Design and Statistical Analyses

Unless noted otherwise, data were analyzed with paired t-test (if distributions are normal) or Mann-Whitney U-test and presented as mean ± SEM. Significance was indicated if P < 0.05. Power analysis: for a t-distribution with mean of μ and standard deviation of 10% of μ, to detect a 10% change in mean with 0.8 power (1-β) we will need an n of 10 animals for each condition. Images for both conditions (open vs. occluded APC) were taken from the same animal. Two (2) – 4 sections were stained for each condition and protein. Two (2) images were taken for each section. Mean values were calculated for one animal. The N numbers reported in the results and figure legends represent the number of animals.

## Results

### Sensory Deprivation Induced Plasticity of Excitatory and Inhibitory Synapses in the Anterior Piriform Cortex

**FIGURE 1:**
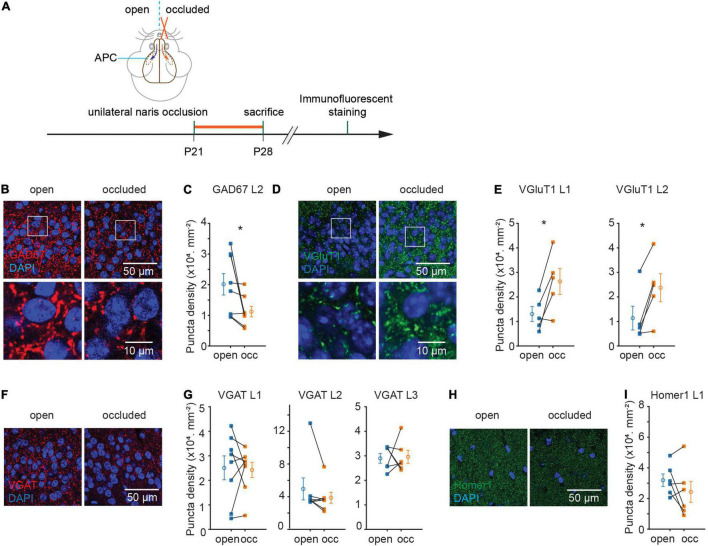
Sensory deprivation induced structural plasticity of excitatory and inhibitory synapses in anterior piriform cortex (APC). **(A)** Timeline of experiment. Unilateral naris occlusion was performed in P21 wild type mice. 7 days after naris occlusion, mice were sacrificed for further immunostaining. **(B)** Representative images of GAD67 puncta in layer 2 of APC. **(C)** Quantification of GAD67 puncta density in layer 2 of APC (p = 0.04, N = 8). **(D)** Representative images of VGluT1 puncta in layer 2 side of APC. **(E)** Quantification of VGluT1 puncta density in layer 1 and layer 2 of APC. (L1: p = 0.03, N = 5; L2: p = 0.02, N = 5). **(F)** Representative images of VGAT puncta in layer2 of APC. **(G)** Quantification of VGAT puncta density in APC (L1: p = 0.84, N = 8; L2: p = 0.22, N = 7; L3: p = 0.87, N = 6). **(H)** Representative images of Homer1 puncta in layer 1 of APC. **(I)** Quantification of Homer1 puncta density in layer 1 of APC (p = 0.23, N = 6). N-number represents the total number of animals used in experiments. n = 3 repeats of experiments were performed. Mann-Whitney test was used for VGAT puncta density in L2. Paired t-test was used for the rest of the datasets. (occ: occluded).

To examine the effects of sensory experience in the APC, we performed unilateral naris occlusion (NO) in young adult mice (∼P21), waited for 7 days for plasticity to occur, and fixed their brains to examine the neurons and synapses (Figure 1A). The open and occluded sides of the olfactory cortex offered an internal control for any effects that NO induces. We opted to examine GAD67 and VGluT1 expression for inhibitory and excitatory synapses, respectively, as they are expressed in most regions of the brain. NO significantly reduced the density of GAD67 + punctae in L2 [(20.1 ± 3.5 to 11.2 ± 1.7) × 10^3^ mm^–2^, p = 0.04, N = 8; Figures 1B,C]. By contrast, NO significantly increased the density of VGluT1 + punctae in both L1 and L2 (L1: (13.0 ± 3.0 to 26.3 ± 5.3) × 10^3^ mm^–2^, p = 0.03, N = 5; L2: (11.4 ± 4.8 to 23.8 ± 5.7) × 10^3^ mm^–2^, p = 0.02, N = 5; Figures 1D,E). On the other hand, NO did not detectably alter the density of punctae marked by VGAT in L1, 2, 3 or Homer1 in L1 of APC [VGAT, L1: (25.1 ± 4.9 to 24.2 ± 3.1) × 10^3^ mm^–2^, p = 0.84, N = 8, L2: (49.5 ± 13.4 to 38.7 ± 6.8) × 10^3^ mm^–2^, p = 0.22, N = 7, L3: (29.0 ± 2.0 to 29.6 ± 2.6) × 10^3^ mm^–2^, p = 0.87, N = 6; Homer1, L1: (31.9 ± 4.1 to 24.3 ± 6.8) × 10^3^ mm^–2^, p = 0.23, N = 6] (Figures 1F–I). These results suggest that NO induced plasticity of opposite direction on inhibitory vs. excitatory synapses, with a decrease of puncta density marked by GAD67 and an increase of puncta density marked by VGluT1 in APC.

### Sensory Deprivation Induced Selective Plasticity in Parvalbumin Neurons

**FIGURE 2:**
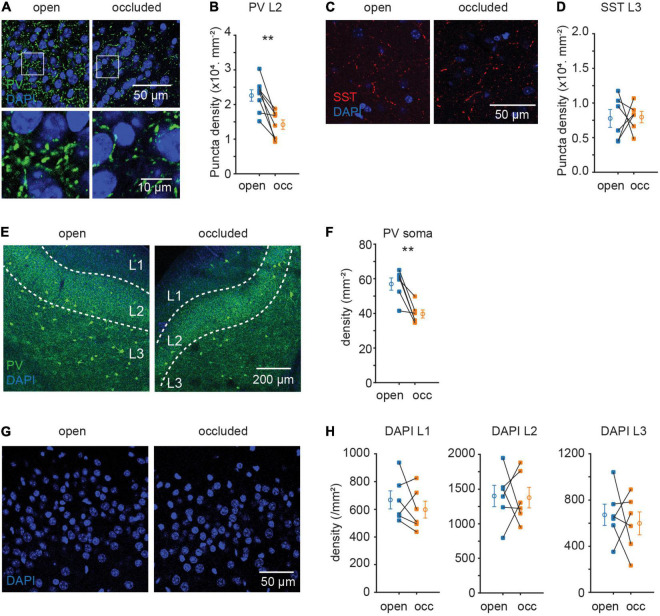
Sensory deprivation induced selective plasticity in PV neurons. **(A)** Representative images of PV puncta in layer 2 of APC. **(B)** Quantification of PV puncta density in layer 2 of APC. (p = 0.002, N = 8) **(C)** representative images of SST puncta in layer 3 of APC. **(D)** Quantification of SST puncta density in layer 3 of APC. (p = 0.93, N = 6) **(E)** representative images of PV neuron in APC. **(F)** Quantification of PV neuron density in APC. (p = 0.008, N = 6) **(G)** representative images of DAPI in layer 2 of APC. **(H)** Quantification of cell density in APC. (L1: p = 0.356, N = 6; L2: p = 0.921, N = 6; L3: p = 0.638, N = 6). n = 3 repeats of experiments were performed. Paired t-test was used for all datasets.

Parvalbumin and SST interneuronal cell bodies reside mostly in L2/3 and are two non-overlapping subtypes of interneurons with sizeable population in APC (Suzuki and Bekkers, 2010). As PV and SST circuits target different subcellular locations on principal neurons, would they be regulated differentially? Immunofluorescent staining against these two markers revealed differential plasticity in these interneuron subtypes: NO significantly reduced the density of PV + but not SST + punctae (PV, L2: (22.6 ± 1.7 to 14.2 ± 1.4) × 10^3^.mm^–2^, p = 0.002, N = 8, Figures 2A,B; SST, L3: (7.8 ± 1.3 to 8.0 ± 0.8) × 10^3^.mm^–2^, p = 0.93, N = 6; Figures 2C,D). The reduction in PV puncta density was accompanied by a reduction in PV soma density (57.0 ± 3.5 to 39.7 ± 2.3.mm^–2^, p = 0.008, N = 6; Figures 2E,F). The reduction in PV + soma density is unlikely to be due to neuronal death induced by NO, as NO did not detectably alter the overall density of cells marked by DAPI (L1: p = 0.356, N = 6; L2: p = 0.921, N = 6; L3: p = 0.638, N = 6; Figures 2G,H). A possible explanation for reduced PV soma density following NO is a decrease in PV protein expression resulting them in “disappearing” from the sections. Overall, these results suggest that sensory deprivation induced selective plasticity in PV but not SST interneuron subtype in APC.

### Sensory Deprivation Upregulated Tnf mRNA Expression in Anterior Piriform Cortex

Our previous results showed that NO altered excitatory and inhibitory synapses and neurons in a manner that is consistent with homeostatic plasticity. It remains unknown, however, whether these are independent events or that there is an external signal that coordinates this regulation.

**FIGURE 3:**
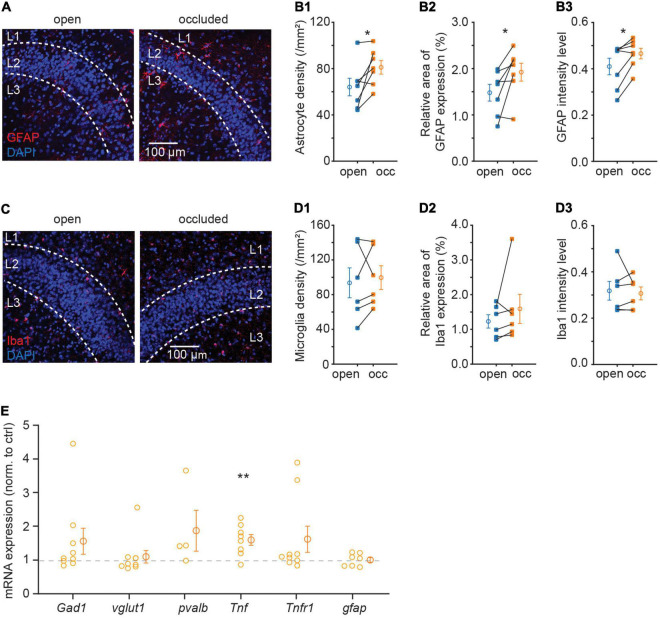
Sensory deprivation increased astrocyte density and expression of TNF-α in APC. **(A)** Representative images of GFAP in APC. **(B)** Quantification of astrocyte density **(B1)**, GFAP relative area of GFAP expression percentage **(B2)** and mean intensity **(B3)** in APC. (B1: p = 0.037, B2: p = 0.038, B3: p = 0.031, N = 7) **(C)** representative images of Iba1 in APC. **(D)** Quantification of microglia density **(D1)**, Iba1 relative area of Iba1 expression percentage **(D2)** and mean intensity **(D3)** in APC. (D1: p = 0.599, D2: p = 0.313, D3: p = 0.676, N = 6) **(E)** relative mRNA expression of Gad1, vglut1, pvalb, Tnf, Tnfr1, and gfap in the occluded side; foldchange is normalized to open side (p = 0.185, N = 9; p = 0.601, N = 9; p = 0.246, N = 4; p = 0.008, N = 8; p = 0.149, N = 9; p = 0.953, N = 7). n = 3 repeats of experiments were performed in **(A–D)**; n = 2–4 repeats of experiments were performed in **(E)**. Mann-Whitney test was used for GFAP mean intensity and Iba1 relative area of Iba1 expression percentage. Paired t-test was used for the rest of the datasets.

In the central nervous system, immune molecules are not only responsible for immune response under pathological condition but also important in regulating normal synaptic function (Vitkovic et al., 2000). Cytokines in the brain are mostly expressed and secreted by glial cells (Alyu and Dikmen, 2017). Considering the importance of immune molecules in synaptic plasticity, we first examined glial cell expression after NO, including astrocytes and microglia, which are responsible for secreting most immune molecules. We examined the changes in astrocytes marked by the cytoskeletal protein, GFAP, and microglia marked by microglia-specific calcium-binding protein, Iba1. NO significantly upregulated GFAP protein expression as indicated by an increase in density and intensity (density: 64.03 ± 7.53 to 81.13 ± 5.93.mm^–2^, p = 0.037, N = 7; intensity: 0.41 ± 0.04 to 0.47 ± 0.02, p = 0.031, N = 7; Figures 3A,B). However, this increase in GFAP signal was not accompanied by a change in Iba1 (density: 93.61 ± 17.23 to 106.80 ± 14.34 mm^–2^, p = 0.599, N = 6; intensity: 0.32 ± 0.04 to 0.32 ± 0.03, p = 0.676, N = 6; Figures 3C,D). These results showed that astrocytes, but not microglia, proliferated following NO. However, it is unclear which molecular process mediates synapse regulation after NO-induced astrocytic proliferation. To determine the possible molecular mediators of this plastic regulation of E-I synaptic density ratio, we used quantitative RT-PCR to examine the relative mRNA expression levels of several synaptic and immune genes in APC. Surprisingly, there was a significant upregulation of Tnf mRNA expression, but not in five other mRNAs assayed (Gad1, p = 0.185, N = 9; vglut1, p = 0.601, N = 9; pvalb, p = 0.246, N = 4; Tnf, p = 0.008, N = 8; Tnfr1, p = 0.149, N = 9; gfap, p = 0.953, N = 7; Figure 3E). TNF-α expressed by astrocytes has been shown to mediate postsynaptic receptor surface expression and synaptic strength (Beattie et al., 2002). Hence, the upregulation in TNF-α mRNA, consistent with increased GFAP protein expression, could be critical in regulating excitatory and inhibitory synapse expression.

### Deletion of Tnf Gene Prevented Plasticity of Excitatory and Inhibitory Synapses

**FIGURE 4:**
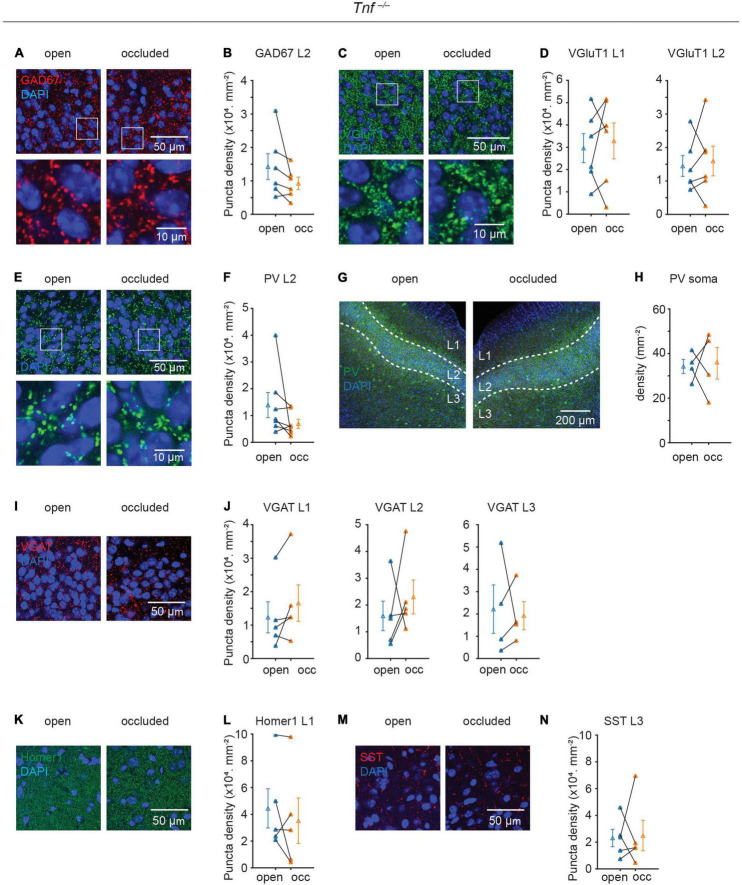
Plasticity of excitatory and inhibitory synapses induced by sensory deprivation is abolished in Tnf^–/–^ mice. **(A)** Representative images of GAD67 puncta in layer 2 of APC. **(B)** Quantification of GAD67 puncta density in layer 2 of APC. (p = 0.148, N = 6) **(C)** representative images of VGluT1 puncta in layer 2 of APC. **(D)** Quantification of VGluT1 puncta density in layer 1 and layer 2 of APC. (L1: p = 0.661, N = 6; L2: p = 0.703, N = 6) **(E)** Representative images of PV puncta in layer 2 side of APC. **(F)** Quantification of PV puncta density in layer 2 of APC. (p = 0.156, N = 7) **(G)** representative images of PV neuron in APC. **(H)** Quantification of PV neuron density in APC. (p = 0.885, N = 4) **(I)** representative images of VGAT puncta in layer 2 of APC. **(J)** Quantification of VGAT puncta density in layer 2 of APC (L1: p = 0.15, N = 5; L2: p = 0.5, N = 5; L3: p = 0.81, N = 5). **(K)** Representative images of Homer1 puncta in layer 1 of APC. **(L)** Quantification of Homer1 puncta density in layer 1 of APC (p = 0.41, N = 5). **(M)** Representative images of SST puncta in layer 3 of APC. **(N)** Quantification of SST puncta density in layer 3 of APC (p = 0.89, N = 5). n = 2– 3 repeats of experiments were performed. Mann-Whitney test was used for PV puncta density in L2. Paired t-test was used for the rest of the datasets.

Although TNF-α is known to regulate both AMPA and GABA_A_ receptors in hippocampal neurons in culture (Altimimi and Stellwagen, 2013; Pribiag and Stellwagen, 2013), its role in regulating excitatory and inhibitory gene expression is unclear. Since we showed that TNF-α was increased following NO, we, therefore, repeated the experiments described above in the Tnf^–/–^ mouse to examine the role of the TNF-α. Strikingly, in the Tnf^–/–^ mouse, all of the plastic phenomena were completely abolished: NO was no longer able to induce plastic changes in GAD67, VGluT1, PV synapse, and PV soma [GAD67, L2: (14.3 ± 3.9 to 9.3 ± 1.9) × 10^3^ mm^–2^, p = 0.148, N = 6; VGluT1, L1: (29.6 ± 6.5 to 32.8 ± 8.1) × 10^3^ mm^–2^, p = 0.661, N = 6, L2: (14.5 ± 3.1 to 16.0 ± 4.4) × 10^3^ mm^–2^, p = 0.703, N = 6; PV puncta, L2: (13.9 ± 4.7 to 6.9 ± 1.7) × 10^3^ mm^–2^, p = 0.156, N = 7; PV soma: 34.2 ± 3.2 to 35.6 ± 7.1 mm^–2^, p = 0.885, N = 4; Figures 4A–H]. GAD67 puncta density between WT and Tnf**^–/–^** mice was similar in the open side (WT open side GAD67 L2: (20.1 ± 3.5) × 10^3^.mm^–2^, n = 8; Tnf**^–/–^** open side GAD67 L2: (14.3 ± 3.9) × 10^3^ mm^–2^, n = 6; p = 0.287). Additionally, in the Tnf^–/–^ mouse, we did not observe alterations in the expression of VGAT, Homer1, or SST, in a similar manner as in the WT mice (VGAT, L1: (12.3 ± 4.6 to 16.6 ± 5.4) × 10^3^ mm^–2^, p = 0.15, N = 5, L2: (15.9 ± 5.5 to 23.0 ± 6.4) × 10^3^ mm^–2^, p = 0.5, N = 5, L3: (22.2 ± 10.9 to 19.2 ± 6.3) × 10^3^ mm^–2^, p = 0.81, N = 4; Homer1, L1: (44.4 ± 14.7 to 35.2 ± 17.0) × 10^3^ mm^–2^, p = 0.41, N = 5; SST, L3: (23.1 ± 6.5 to 24.9 ± 11.4) × 10^3^ mm^–2^, p = 0.89, N = 5; Figures 4I–N). These results strongly suggest that TNF-α is a key inducer of the plastic changes in gene expression of excitatory and inhibitory synapses.

## Discussion

Cytokines secreted by glial cells are increasingly recognized to be important mediators of neuroglial interactions, particularly during experience-dependent plasticity. Here we show that activity deprivation in the form of NO induces structural plasticity of certain excitatory and inhibitory markers in an opposite manner: NO increases VGluT1 expression and decreases GAD67 and PV expression. Importantly, genetic deletion of Tnf entirely prevented these plastic regulations. Overall, our findings indicate that TNF-α is a critical orchestrator in modulating sensory deprivation-induced plasticity.

Anterior piriform cortex, as one of the first cortical destination of olfactory output, is only two synapses away from the periphery and its function is highly modulated by ongoing sensory experience (Wilson and Sullivan, 2011). Therefore, APC offers a suitable model to study the neural and genetic mechanisms of how experience regulates neural circuit plasticity. Scaling of synapses and balancing of E-I ratio are common features of homeostatic plasticity (Turrigiano et al., 1998). Here we showed that sensory deprivation induced plasticity of synapses in a manner that is consistent with homeostatic regulation, with an increase of excitatory synaptic density and a decrease of inhibitory synaptic density. One limitation of our study is that synaptic protein expression indicates only structural but not functional change. Our group has recently shown that NO increased the conductance of spontaneous and evoked IPSCs in a subtype of principal neuron in APC (Jiang et al., 2021). However, our current study shows that density of inhibitory synapses, especially PV^+^ synapses, are decreased after NO. Possible reasons for this discrepancy between anatomy and physiology could be: First, not all structural synapses are functional (or connected) in synaptic transmission; Second, synaptic conductance can be influenced by properties such as release probability (Fatt and Katz, 1952), transmitter clearance (Liu et al., 1999) and postsynaptic GABA_A_R expression (Chu and Hablitz, 1998); Third, in Jiang et al’s. (2021) paper, much of the functional measurements were focused on recurrent inhibition. However, in this study, both synapses of afferent and recurrent inputs to principal neurons are quantified in L2. Fourth, here we used mice with age ranging from p21–p28 while Jiang et al. used mice with age ranging from p42 to p45. The age difference may affect the plasticity of synapse after NO. Furthermore, NO selectively enhanced PV, but not SST, inhibitory conductance (Jiang et al., 2021). These results are consistent with our present study, where NO induced selective plasticity in PV but not SST neuron. Although puncta density could be underestimated since it is quantified from two dimensional images, our results raise the possibility that perisomatic but not dendritic inhibition mediates homeostatic regulation after sensory deprivation. Overall, it is still unknown what is the physiological effect of TNF-α on functional E-I ratio after sensory deprivation in APC. The effect of TNF-α can be further explored by recording from slices of Tnf**^–/–^** mouse before and after applying exogenous TNF-α.

Using transgenic mice, we demonstrated that regulation of excitatory and inhibitory synapses following NO is completely abolished in *Tnf***^–/–^** mice, indicating that TNF-α is a key factor in experience-dependent plasticity (Figure 4). An unsolved question is what contributes to the increase of TNF-α level following NO in wild type mice. *Tnf* mRNA level, consistent with TNF-α protein level, are both increased in dentate gyrus slice after entorhinal denervation (Becker et al., 2013) and in nucleus accumbens after cocaine treatment (Lewitus et al., 2016). Both *Tnf* mRNA and TNF-α immunofluorescence are colocalized with GFAP-immunofluorescence, suggesting astrocytes are a major source of TNF-α (Becker et al., 2013). Astrocyte-conditioned media exhibits the same effect as exogenous TNF-α in increasing surface AMPAR expression and miniature EPSC (mEPSC) frequency, which is blocked by co-application of a soluble form of TNFR1, suggesting that TNF-α from astrocytes increases surface expression of AMPAR and synaptic strength (Beattie et al., 2002). Considering that astrocytes proliferated in the APC after NO (Figure 3), it is probable that astrocytes are responsible for the increase of TNF-α level that mediates homeostatic regulation induced by sensory deprivation.

Besides modulating postsynaptic receptor surface expression, TNF-α may also regulate presynaptic neurotransmitter release. TNF-α treatment increased mEPSC frequency (Beattie et al., 2002) and decrease mIPSC frequency (Pribiag and Stellwagen, 2013) in hippocampal neurons. mEPSC or mIPSC frequency could be controlled by vesicular glutamate or GABA concentration in the presynaptic terminal, respectively. TNF-α increased spontaneous EPSC frequency by increasing transient receptor potential subtype V1 (TRPV1)-mediated glutamate release in presynaptic terminal (Park et al., 2011). Our results that TNF-α regulates excitatory and inhibitory synaptic density in the presynaptic terminal are consistent with these studies. We showed that VGluT1, but not Homer1 puncta density is increased after NO. VGluT1 is associated with presynaptic glutamate transport and Homer1 is associated with post-synaptic scaffolding. It is possible that TNF-α regulates excitatory synaptic transmission by altering presynaptic glutamate release instead of postsynaptic spine formation. VGAT localizes to synaptic vesicles in glycinergic as well as GABAergic neurons (Chaudhry et al., 1998). Our results that GAD67, but not VGAT is decreased after NO, suggests that TNF-α may regulate overall GABA concentration. Further experiments are required to elucidate the mechanisms by which TNF-α regulates the presynaptic properties of excitatory and inhibitory synapses.

The requirement of TNF-α in homeostatic synaptic plasticity is time-dependent. Synaptic scaling is a cumulative process which is observed after acute application (4 h) of TTX (Ibata et al., 2008), while TNF-dependent scaling was observed only after chronic activity blockade (24 h) (Stellwagen and Malenka, 2006). Only prolonged inhibition of TNF-α signaling by addition of soluble TNFR can prevent late-stage synaptic scaling (24 to 48 h) following activity deprivation in rat cortical neurons (Steinmetz and Turrigiano, 2010). Entorhinal denervation induced synaptic upscaling at both early (1–2 days post lesion) and late phase (3–4 days post lesion) in dentate gyrus, but TNF-α only mediates the late phase component (Becker et al., 2013). While we did not examine shorter time periods than 7 days, our results that *Tnf* mRNA and astrocytic activation are elevated at 7 days after NO suggest that TNF-α is required for long-term homeostatic regulation of synaptic density in APC *in vivo*.

In addition to *in vitro* studies, other reports have revealed the role of TNF-dependent plasticity in sensory systems of intact animals. In layer 5 of the barrel cortex, a slower potentiation in response to unilateral whisker trimming deprivation in principal neurons is abolished in *Tnf***^–/–^** mice (Greenhill et al., 2015). In the primary auditory cortex, a multiplicative increase in synaptic strength after 3 days of chronic hearing loss was impaired in *Tnf***^–/–^** mice (Teichert et al., 2017). In developing visual cortex, TNF-α is required for the late potentiation phase of plasticity after monocular deprivation (Kaneko et al., 2008). While the critical role of TNF-α has been shown in many sensory systems, little work was done in the olfactory system. The circuit organizational principles in the APC are closer to other regions such as the hippocampus and prefrontal cortex in that the APC has extensive recurrent connections and is without topographic mapping. This is partly why the APC can perform functions like context-dependent memory storage and retrieval (Haberly, 2001). Moreover, the APC is largely innervated by projections from the ipsilateral olfactory bulb, making it quite different from other sensory cortices like primary auditory and visual cortices, which are innervated by bilateral connections. These features that are unique to the APC make it worthwhile to study the effects of TNF-α in APC because the published phenomena in the auditory and visual systems are not immediately applicable to the olfactory system. Here we show that genetic deletion of *Tnf* abolished the plastic changes in VGluT1, GAD67, and PV protein expression. While we did not observe any changes in gene expression following NO in *vglut1*, *Gad1*, and *pvalb* mRNA, we did not measure the total protein expression. It is possible that the reduction in GAD67 and PV puncta density in L2 is due to altered trafficking and/or clustering of these proteins (Kanaani et al., 2010). How can TNF-α regulate a diverse population of neurons including both excitatory and inhibitory neurons? TNFR1 predominantly binds to soluble TNF-α and is constitutively expressed on most cells of the body (Becker et al., 2015). A survey for TNFR1 expression in the Allen Mouse Brain Atlas showed that *TNFR1* mRNA is positively expressed in neurons in L2 (mainly glutamatergic) and L3 (glutamatergic and GABAergic)^1^. Hence, it is possible that both glutamatergic and GABAergic neurons are receptive to the effects of secreted TNF-α. A parsimonious explanation is that TNF-α is a key astrocytic molecule induced during activity deprivation that orchestrates the various plastic events occurring in the presynaptic terminals of glutamatergic and GABAergic neurons. Our study provides an insight into the role of TNF-α in regulating plasticity in olfactory cortex. The signaling mechanism, however, by which TNF-α exerts control on both neuron types remains to be deciphered.

**FIGURE 5:**
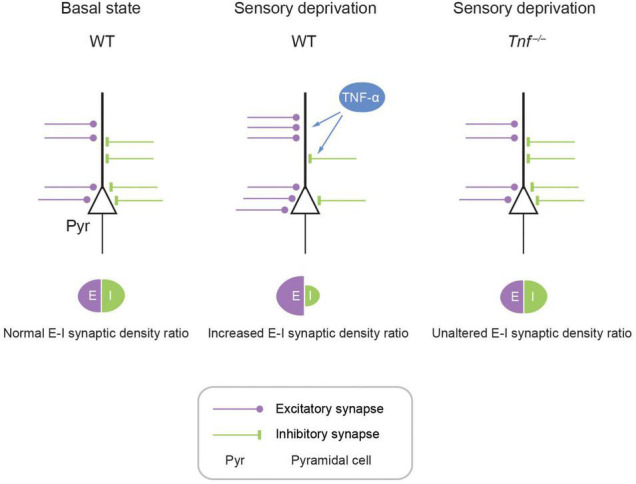
Schematic model showing TNF-α mediates experience-dependent modulation of synaptic plasticity of structural excitatory-inhibitory ratio in APC. In the APC, E-I synaptic density ratio is determined by local neural activity, which in turn is primarily determined by the upstream activity in the OB. Fluctuating activity levels induce plasticity in the APC. For example, hypo-activity in the form of sensory deprivation can increase excitation while decreasing inhibition, achieving an enhancement of E-I synaptic density ratio. This augmentation of E-I synaptic density ratio is mediated by astrocytic TNF-α, as evidenced by the complete abolishment of this plasticity in the *Tnf*^–/–^ mouse.

Taken together, we propose a model where deprivation of olfactory sensory activity in the APC induces homeostatic plasticity of excitatory and inhibitory synapses and neurons such that the overall E-I synaptic density ratio is increased in the network (Figure 5). To the best of our knowledge, this study represents the first report showing that TNF-α modulates experience-dependent plasticity in the mammalian olfactory cortex. This is important as APC is a primary sensory cortex that also exhibits associative properties such as context-dependent retrieval and storage of memory due to dense recurrent excitatory inputs and robust long-term synaptic plasticity (Haberly, 2001; Best and Wilson, 2003; Poo and Isaacson, 2007; Franks et al., 2011). Hence, neurons in APC can process odor information depending on context, which implements homeostatic regulation of E-I balance *via* the TNF-α system. We believe that any new scheme for modeling APC circuit function and computation should include TNF-α-mediated deprivation-induced plasticity.

## Data Availability Statement

The original contributions presented in the study are included in the article/Supplementary Material, further inquiries can be directed to the corresponding author.

## Ethics Statement

The animal study was reviewed and approved by Animal Research Ethics Sub-Committee of City University of Hong Kong and Department of Health of Hong Kong SAR government.

## Author Contributions

AG performed all experiments including naris occlusion, immunofluorescent staining, quantitative PCR, and analyzed data. AG and CGL designed the research, wrote and edited the manuscript. Both authors contributed to the article and approved the submitted version.

## Conflict of Interest

The authors declare that the research was conducted in the absence of any commercial or financial relationships that could be construed as a potential conflict of interest.

## Publisher’s Note

All claims expressed in this article are solely those of the authors and do not necessarily represent those of their affiliated organizations, or those of the publisher, the editors and the reviewers. Any product that may be evaluated in this article, or claim that may be made by its manufacturer, is not guaranteed or endorsed by the publisher.

## Footnotes

1
https://mouse.brain-map.org/experiment/show/2689
